# An Investigation of the Influence of Viscosity and Printing Parameters on the Extrudate Geometry in the Material Extrusion Process

**DOI:** 10.3390/polym15092202

**Published:** 2023-05-06

**Authors:** Shahriar Bakrani Balani, Hossein Mokhtarian, Tiina Salmi, Eric Coatanéa

**Affiliations:** 1 Faculty of Engineering and Natural Sciences, Tampere University, 33720 Tampere, Finland; 2 Faculty of Information Technology and Communication Science, Tampere University, 33720 Tampere, Finland

**Keywords:** additive manufacturing, material extrusion process, fused filament fabrication, finite element modeling, computational fluid dynamic, numerical simulation, polylactic acid, PLA, two-phase flow modeling, level-set

## Abstract

The material extrusion process is one of the most popular additive manufacturing processes. The presence of porosity in the MEX printed parts, which ultimately deteriorates the mechanical properties, is one of the main drawbacks of the MEX process. The porosity in the structure is related to the shape of the adjacent beads and overlapping during the material deposition. Due to the deposition nature of the MEX process, the porosity cannot be entirely removed from the printed parts. Understanding the influence of process parameters on material deposition and the rheological properties is crucial to improving the quality of the final product. In this study, the two-phase-flow numerical approach with the level-set equations has been used for the first time to model the material deposition on the moving platform in 3D. The influence of the viscosity and printing parameters, including travel speed, inlet velocity, viscosity, nozzle diameter, and layer height, on the width of the deposited bead has been investigated. The simulation results are validated against experimental measurements with an average error of 5.92%. The width measured by the experimental study shows good agreement with the results of the numerical simulation. The comparison between the results of the 3D numerical simulation and 2D simulation reveals that the 2D simulation is not appropriate and accurate enough to predict the geometry of the deposited bead with the given set of parameter settings. The key novelty of this research paper is the application of the level-set method in a 3D context for material deposition on a moving substrate.

## 1. Introduction

According to ISO/ASTM 52900:2015, material extrusion (MEX) refers to an “additive manufacturing process in which the material is selectively dispensed through a nozzle or orifice” [1]. Fused filament fabrication (FFF) is a widely used MEX-based process. The popularity of the FFF process is arguably due to its cost-effectiveness and easy accessibility. The FFF process enables the printing of a wide range of polymers: from polylactic acid (PLA) and acrylonitrile butadiene styrene (ABS) as the most popular material to high-performance materials such as polyaryl ether ketone (PAEK) family. The simplicity and rapidity of the process make the MEX process convenient for not only rapid prototypes but also manufacturing semi-final and final parts [2]. However, poor mechanical strength and lack of dimensional accuracy are among the drawbacks of the MEX process [3]. In the MEX process, the parts are manufactured by successive deposition of the beads according to a predefined trajectory. The porosity in the structure appears due to the shape of the adjacent beads during material deposition. Consequently, the parts printed by the MEX process often have a relatively high porosity ratio due to the nature of the material deposition strategy [4,5]. The parts with a higher porosity ratio have lower mechanical properties [6]. Inconsistency in the bead size (width and height) leads to an increase in porosity. Although the porosity cannot be entirely removed from the printed parts, understanding the influence of the printing parameters on the geometry of the deposited bead is essential to controlling the geometry of the deposited bead and adjusting them according to the desired geometry [6]. Hence, controlling the printing parameters is also essential in order to avoid excess material deposition to ensure uniformity of the width of the deposited bead. Adhesion/bonding between the deposited beads is one of the most crucial properties influencing the mechanical properties of printed parts [7]. Improving the quality of the printed parts entails suitable process parameter selection. Suitable process parameters are identified via experimental and/or numerical simulation approaches. The experimental approach provides empirical knowledge based on the observations and measurements for given hardware settings. Numerical simulation is a cost-effective and environmentally friendly alternative that enables the theoretical studying of process parameters’ influence. Numerical simulations are advantageous especially when measurements are time-consuming, costly, and require additional sensors [8].

It has been proven in the literature that a numerical simulation is an adequate approach to effectively study the MEX process properties [9]. Rashid and Koç reviewed the numerical simulation techniques used in the FFF process [9]. They have categorized the research developments based on the type of performed numerical analysis. Studying the properties of the MEX process via numerical simulation entails solving multiphase fluid problems. Several methods are proposed for multiphase numerical modeling of the fluids. Level-set (LS) [10], volume-of-fluid (VOF) [11], and phase-field (PF) [12] are among the most important modeling approaches for two-phase flow (TPF) models. Applying the LS method allows for more accurate computation of curvature, and therefore, it results in better smoothness of discontinuities near interfaces. In contrast, the VOF method is not able to compute accurate and smooth curvature near the interfaces since the VOF is performed as a step function [13]. However, as reported in the literature, the LS method is more prone to numerical errors compared with the VOF method [13]. The numerical error is likely to occur when the interfaces experience severe stretching (see [13]). Several authors proposed coupling the two above-mentioned modeling approaches to tackle the limitation of each individual method [14,15,16,17]. Sussman et al. proposed a coupled LS and VOF approach (CLSVOF) for TPF modeling to increase the accuracy of the results when the effect of surface tension energy and topology is important [14]. Their work demonstrated that the CLSVOF approach exhibits more accurate results compared with when LS and VOF are separately applied. Xia et al. proposed a computational model for modeling the shape of the deposited bead in the MEX process by considering the viscoelastic properties of the polymer [18]. In this study, material flow from the nozzle and deposition on a substrate or previously deposited beads are modeled. The proposed method for tracking the polymer interface with the air is based on the front-tracking/finite-volume method. Xia et al. have successfully modeled the properties of the MEX process, including fluid flow, heat transfer, and viscoelastic behavior of the deposited bead. Additionally, they have modeled the die swelling of the extrudate in the MEX process [18]. Due to the modeling complexity, there are no abundant studies on the bead’s shape on the moving platform. Modeling the bead geometry requires mathematically identifying the polymer/air interface and hence considering the properties influencing the shape of the deposited bead.

Computational fluid dynamics (CFD) and the VOF approach enable us to model the deposition of the bead on the platform and its heat transfer [19]. Serdeczny et al. have modeled the deposition of the bead on the platform using commercial numerical simulation ANSYS Fluent R18.2 software (Anys, Canonsburg, PA, USA) [20]. The results of their numerical simulation are validated against experimental measurements. Furthermore, they have shown that the CFD approach could be used to model several beads deposited together [21]. The same authors have studied the effect of motion planning along the shape edges using VOF numerical simulation [22]. The same modeling approach has been used by other authors to numerically model the extrusion die process [23]. In this modeling approach, momentum, continuity, and energy equations are solved to model the fluid flow. Pricci et al. studied the process variables, such as mass flow rate, melting profile, and pressure profile, in the pellet-based additive manufacturing process [24]. The objective of this study was to determine the screw velocity to control the desired mass flow rate. Pricci et al. have proposed a mathematical model to describe the process from solid pellets to melt. The mathematical approach has been validated with numerical simulation and an experimental study [24]. Their numerical simulation is based on the CFD approach. Pham et al. also studied the rheological properties of PLA in the MEX process using the CFD numerical simulation [25]. The melting profile, pressure drop, and viscosity in the nozzle have been determined using the CFD-VOF approach and Ansys software [20]. Gharehpapagh et al. experimentally studied the concept of dynamically changing the width of the bead in the MEX process [26]. They investigated the bead geometry with a rectangular orifice. They have concluded that bead geometry could be controlled by controlling the orientation of the rectangular orifice [26].

To the best of our knowledge, there is no study in the literature on the 3D modeling of material deposition using LS equations. The objective of this article is to use the TPF-LS approach to model the influence of printing parameters such as layer height, nozzle diameter, inlet velocity, and travel speed on the shape of the deposited bead. In practice, users do not define the material inlet velocity in parameter settings of slicing software. Therefore, an equation is proposed to determine the inlet velocity in the nozzle according to the printing parameters, filament diameter, and nozzle diameter. The geometry of the deposited bead derived from numerical simulations is compared with experimental printed beads. Since 3D modeling using the LS equations is computationally demanding, a comparison between 2D numerical simulation and 3D is carried out with the same inputs and boundary conditions to evaluate whether 2D modeling would result in the same output.

**Figure 1:**
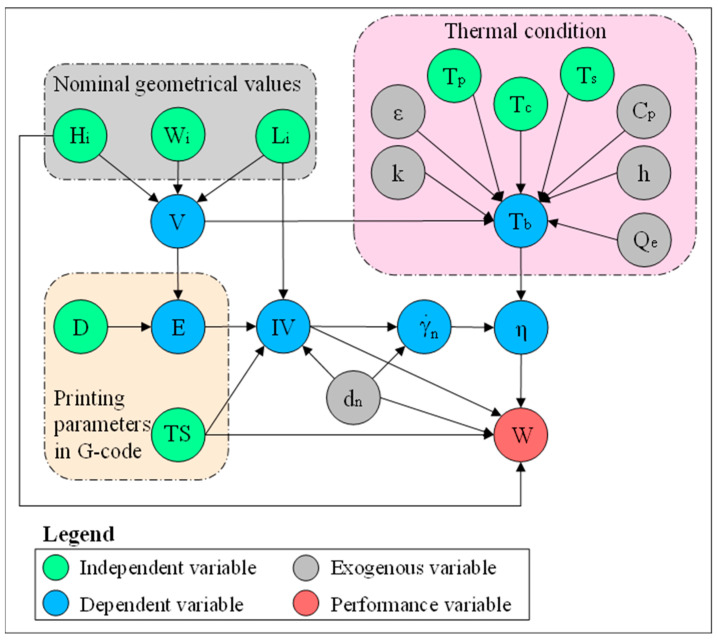
Representation of an oriented graph—parameters influencing the bead width in the MEX process.

To visualize the set of parameters and variables involved in this study and their relationships, a colored directed graph is developed using the dimensional analysis conceptual modeling (DACM) framework [27], shown in Figure 1. Causal graphs are oriented graphs in which the nodes represent the variables/parameters, and the orientation of edges shows the causality relationship between the variables. Note that this oriented graph is not unique; different similar graphs can be developed depending on the study focus, required details, adopted assumptions, process, and intended objectives. To build a causally oriented graph, the DACM framework classifies the variables into four main color-coded categories. The independent variables (shown in green) are input process parameters or independent design variables that can be freely set by the designer or modeler. The independent variables are not influenced by any other variables in the system of interest. In the scope of this research, the independent variables are the variables describing nominal geometrical values of the extrudate, thermal conditions, and other process parameters. The variables describing nominal geometrical values of the extrudate are layer height (H_i_), intended initial width of the bead (W_i_), and intended length of the bead (L_i_). The nominal geometrical parameters and slicer printing parameters are used to calculate the filament inlet velocity (IV). Thermal condition is defined by different temperature points set on the machine, namely, printing temperature (T_p_), substrate temperature (T_s_), and the temperature of the printing chamber (T_c_). Nozzle travel speed (TS) and filament diameter (D) are also independent variables that define filament inlet velocity (IV). The exogenous variables (shown in grey) refer to the variables outside the borders of the system or scope of the study. The exogenous variables are the variables that are kept fixed or imposed on the system. The material properties as well as the nozzle-related variables are considered exogenous variables in the oriented graph. Material properties are considered fixed values in this study as a modeling assumption. Dependent variables (shown in blue) are influenced by other variables, such as exogenous and independent variables. The dependent variables can be controlled indirectly. Among the dependent variables, the inlet velocity (IV) is determined by TS, L_i_, and the extruder increment (E). The extruder increment (E) is the length of the filament entering the extruder for a given length. The extruder increment (E) is determined by slicing software based on D and the required volume of the extrudate (V). Viscosity (η) is influenced by the temperature of the deposited bead (T_b_) and shear rate in the nozzle (${\dot{\gamma }}_{n}$) and is used to determine the width of the deposited bead (W) together with other parameters shown in the oriented graph. In the current research, viscosity calculated by Carreau–Yasuda model is used instead of constant viscosity. The performance variables (shown in red) are the ultimate objective of the design and modeling task. The performance variables are selected by the designer or modeler as a performance indicator of the system of interest. In the current study, deposited bead (W) is the performance parameter. This oriented causal graph is used as a knowledge representation tool to describe the influencing parameters in the problem of interest.

The remainder of this paper is structured as follows: Section 2 describes the material characterization and overall methodology applied in this research. More specifically, Section 2 focuses on the material characterization of PLA and describes the experimental study and numerical simulation of material deposition. Section 3 articulates the results of the numerical simulations and experimental validation. This section first discusses the accuracy of the developed model in determining the geometry of the deposited bead width. Comparing the geometry of the deposited bead derived from simulation with the experimental study validates the numerical simulation. The numerical simulation then focuses on the influence of viscosity on the shape of the extrudate (deposited bead). Last, the section compares the results of 2D and 3D numerical simulations. Section 4 concludes the main findings of this research.

## 2. Materials and Methods

### 2.1. Material Characterization

**Figure 2:**
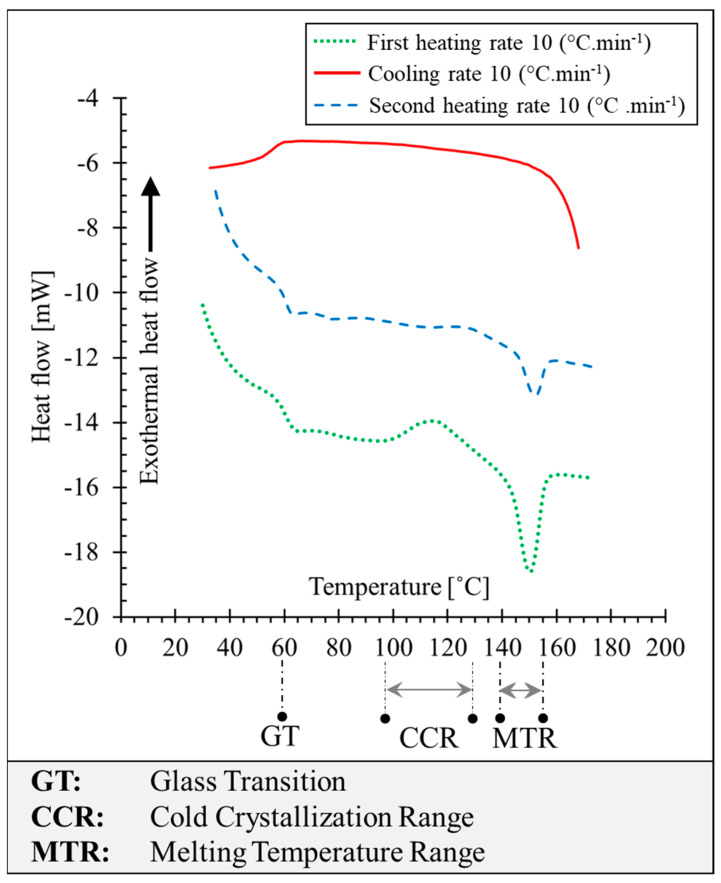
Representation of the differential scanning calorimetry (DSC) thermogram of PLA.

This research was initiated with the characterization of PLA filament manufactured by PRUSA (Prague, Czech Republic). Determining the transition temperatures of polymers is a prerequisite for the viscosity measurement. For the DSC test and parallel plate rheometer test, PLA filament is dried for 24 h at a temperature of 60 °C and stored in a desiccator. The thermal transitions have been experimentally determined using a Q200–TA differential scanning calorimetry (DSC) apparatus (TA Instruments, New Castle, DE, USA) under nitrogen gas flow. The thermogram curves in Figure 2 represent the glass transition of PLA occurring at 60 °C, the melting peak is at around 150 °C, and melting is completed at around 155 °C. Cold crystallization is observed in the first heating ramp (represented in green) starting from approximately 97 °C and ending at about 130 °C with an enthalpy of 13 J·g^−1^. Melting endothermic enthalpy at the first heating ramp is 22 J·g^−1^. No crystallization is observed in the cooling phase, and glass transition takes place at around 55 °C. At the second heating ramp (represented in dashed blue), the glass transition is observed at around 60 °C (See GT marked in Figure 2). A small cold crystallization peak is observed between 125 °C and 140 °C. The melting enthalpy of 5 J·g^−1^ is observed around 155 °C.

The obtained results by the DSC test show that the crystallization kinetics of this grade of PLA is relatively slow compared with other semi-crystalline polymers, such as PEEK, and even other grades of PLA [28]. Furthermore, due to the fast-heating rate (10 °C·min^−1^), macromolecular chains of the polymer do not have time to reorganize and form the crystalline phase. Considering the cooling rate of the polymer melt in the MEX process, printing with this grade of PLA leads to the amorphous structure in the printed samples.

**Figure 3:**
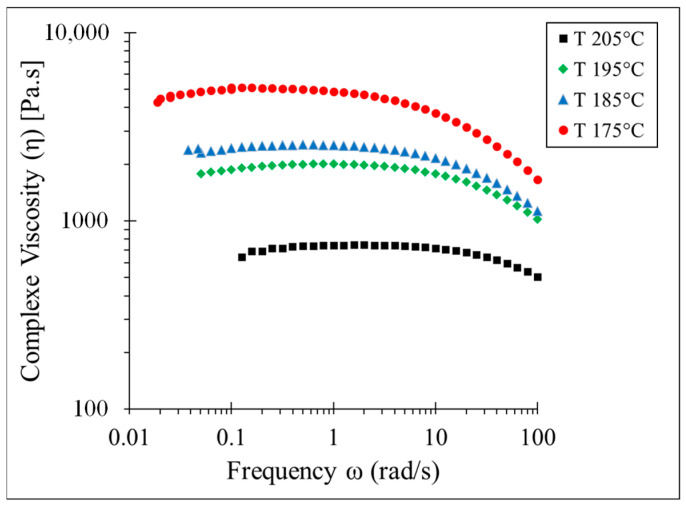
Complex viscosity of PLA at different frequencies, determined experimentally by parallel plate rheometer.

Determination of the viscosity is necessary in order to model the fluid flow. Consequently, we have determined the complex viscosity (η*) using the parallel plate configuration of the rheometer. According to Cox–Merz rule, when the angular frequency is equal to the steady shear rate, complex viscosity, and steady shear viscosity are equivalent. The complex viscosity (η*) of PLA from low frequency (below 0.1 Hz) until high frequency (100 Hz) has been determined at four temperature points above the melting temperature, by using the parallel-plate setting of the ARES rheometer (TA Instruments, New Castle, DE, USA) [29,30]. Figure 3 represents the results of test experiments at four temperature points of 175 °C, 185 °C, 195 °C, and 205 °C. At the low frequency and especially at the higher temperature point (e.g., 205 °C), PLA undergoes thermal degradation. As expected, PLA demonstrates shear-thinning behavior. Increasing the temperature and frequency (shear rate) decreases the complex viscosity. At 175 °C, complex viscosity reaches approximately 5000 Pa·s, while at 205 °C, complex viscosity reaches 600 Pa·s. This represents the dependency of the complex viscosity on the temperature. At proximity to the melting point, the influence of the frequency on the complex viscosity is greater than on the viscosity at a higher temperature. For instance, at 175 °C and at the terminal regime ($\eta_{0}$($\dot{\gamma }\sim 0$)), complex viscosity is about 5000 Pa·s, while at 100 Hz, complex viscosity is below 2000 Pa·s.

In order to implement the viscosity determined by the parallel-plate rheometer in the numerical model, the Carreau–Yasuda viscosity model has been fitted on the viscosity determined at 195 °C. The Carreau–Yasuda model defines viscosity as a function of the shear rate while considering the Newtonian plateau at low shear rates [31]. The Carreau–Yasuda viscosity model is represented in Equation (1):

(1) $$\eta =\eta_{inf}+\left(\eta_{0}-\eta_{inf}\right){\left[1+{\left(\lambda \dot{\gamma }\right)}^{a}\right]}^{\frac{n-1}{a}}$$

where n is the pseudoplasticity index, K is the consistency coefficient, η_0_ is the viscosity of the fluid at zero shear rate, η_inf_ is the viscosity of the fluid at the infinite shear rate, λ is the relaxation time index, a is a dimensionless parameter describing the transition between the first Newtonian plateau and the power law zone, and $\dot{\gamma }$ is the shear rate. Thermoplastics are shear-thinning fluids with pseudoplasticity index (n) below 1, which means when the shear rate increases, viscosity decreases. Table 1 reports the values for the parameters of the Carreau–Yasuda equation for PLA at 195 °C. These parameters are used for the development of the numerical simulation in this paper. The deposition of the extrudate immediately after exiting from the nozzle is also modeled in this paper. The time interval of material deposition is relatively short (0.25 s); thus, the deposited bead does not have enough time to have heat transfer with the environment and as a result, it could be considered a quasi-isothermal condition [5]. Consequently, the influence of the temperature variation on the viscosity is neglected.

To better study the influence of the printing parameters on the shape of the deposited bead, it is necessary to determine the velocity field and shear rate in the nozzle (before exiting material from the nozzle). The velocity field along the diameter of the nozzle can be modeled as a Hagen–Poiseuille flow [30]. The velocity field in the nozzle according to the rheological properties of the polymer and printing parameters are determined using Equation (2) [30]:

(2) $$u\left(r\right)=\frac{3n+1}{n+1}\bar{IV}\left[1-{\left(\frac{r}{{0.5*d}_{n}}\right)}^{\frac{\left(1+n\right)}{n}}\right]$$

where $\bar{IV}$ is the mean inlet velocity of the polymer in the nozzle, r is the radial distance from the center of the nozzle, d_n_ is the diameter of the nozzle, and n is the pseudoplasticity index. Derivation of the velocity field results in the shear rate equation in the nozzle, shown in Equation (3):

(3) $$\dot{\gamma }=\frac{3n+1}{n*0.5*d_{n}}*\bar{IV}*\left[{\left(\frac{r}{0.5*d_{n}}\right)}^{\frac{1+n}{n}-1}\right]$$

Table 2 summarizes the properties and characteristics of the PLA filament used in this research.

### 2.2. Numerical Simulation

Numerical simulation of the material deposition on a moving substrate in the MEX process is carried out using TPF numerical simulation approach with LS equations [32] in COMSOL Multiphysics 6.1 software (COMSOL, Burlington, MA, USA). In this approach, the Navier–Stokes and continuity equations are used to model the flow of the phases (polymer melt and air) [29]. An additional equation is added to the system of equations to track the interface of the two phases. The fluids are considered incompressible, and the polymer flow is considered stokes flow or creeping flow (the inertial term is neglected).

The fluid is considered incompressible fluid; thus, the continuity equation yields Equation (4). The Navier–Stokes equation used in our study is shown in Equation (5), where ρ is the density, u is the fluid velocity, p is the pressure applied to the fluid, μ is the dynamic viscosity of the fluid, and g is the gravity field. F_st_ represents the force resulting from the surface tension and F represents all other external forces. Equation (6) represents the LS equations used in TPF simulation. The parameter φ is the volume fraction, $\varepsilon_{Is}$ defines the interfacial thickness parameter, and γ is the re-initialization parameter. The re-initialization parameter (γ) is considered the maximum or close to the maximum velocity of the fluid in the TPF system to ensure the consistency of the results with the whole simulations. Our empirical tests show that reducing the $\varepsilon_{Is}$ (interfacial thickness) value influences the thickness of the interface between two phases, consequently getting better accuracy between the phases. In TPF simulation, the density and viscosity of each mesh are determined using Equations (7) and (8) according to the volume fraction. Where $\rho_{polymer}$ and $\rho_{air}$ represent the density of polymer and air respectively, $\eta_{polymer}$ and $\eta_{air}$ are the viscosities of the polymer and air.

(4) ∇.u=0

(5) $$\rho \frac{\partial u}{\partial t}=\nabla .\left[-pI+\mu (T)(\nabla u+{\left(\nabla u\right)}^{T})\right]+\rho g+F_{st}+F$$

(6) $$\frac{\partial \varphi }{\partial t}+\nabla .\left(u\varphi \right)=\gamma \nabla .\left(\varepsilon_{Is}\nabla \varphi -\varphi \left(1-\varphi \right)\frac{\nabla \varphi }{\left|\nabla \varphi \right|}\right)$$

(7) $$\rho =\varphi \rho_{polymer}+\left(1-\varphi \right)\rho_{air}$$

(8) $$\eta =\varphi \eta_{polymer}+\left(1-\varphi \right)\eta_{air}$$

**Figure 4:**
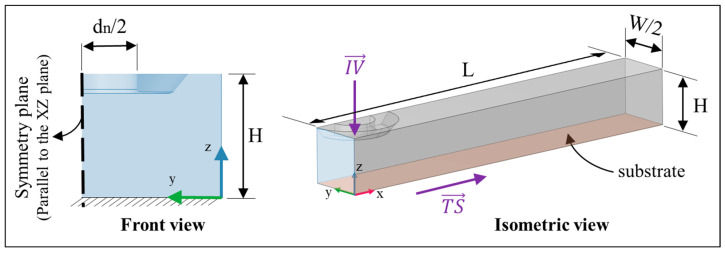
Boundary conditions of the 3D model developed for the material deposition.

Figure 4 represents the boundary conditions for the numerical simulation performed in this study. Polymer melts enter the nozzle through the inlet orifice with the inlet velocity of IV. Travel speed (TS) is assigned to the platform. The contact angle of the polymer melt with the substrate is determined through an experimental study and is considered 30 degrees. In the case of single bead deposition, H_i_ is the distance between the platform and the nozzle. The unnecessary part of the liquefier is neglected; therefore, just the printing nozzle is modeled. In addition to the definition of symmetry in the XY plane, the size of the model is optimized to reduce the computation time. The deposition substrate length is 3.5 mm, and the width is 0.5 mm. The distance between the platform and nozzle is modeled with a rectangular shape, which represents the air phase in the model. The selection of substrate length and width is of particular importance since a large substrate length highly increases the computation time and a short substrate length leads to inaccurate bead geometry determination. The gravity force is applied to the system in the Z direction to consider the effect of the mass of the polymer flow on the system. The influence of viscosity (η), nozzle diameter (d_n_), layer height (H_i_), inlet velocity (IV), and travel speed (TS) is investigated by numerical simulation. Note that contrary to the RepRap printer that the substrate is fixed and the extruder is moving during material deposition, in the TPF numerical simulation extruder is considered fixed while the deposition substrate is moving according to the TS. Re-initialization parameter (γ) value in the LS equation is selected to be 15 mm·s^−1^ and the parameter controlling the thickness is kept as the default value. The generalized minimal residual (GMRES) method is selected for the fluid flow and LS solver. The contact angle between extrudate and substrate is determined through experimental study and set in the numerical simulation.

**Figure 5:**
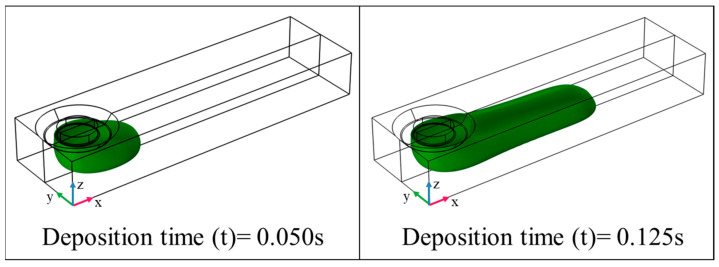
Three-dimensional presentation of the material deposition on the substrate (d_n_ = 0.4 mm, IV = 0.25 mm·s^−1^, TS = 20 mm·s^−1^).

**Figure 6:**
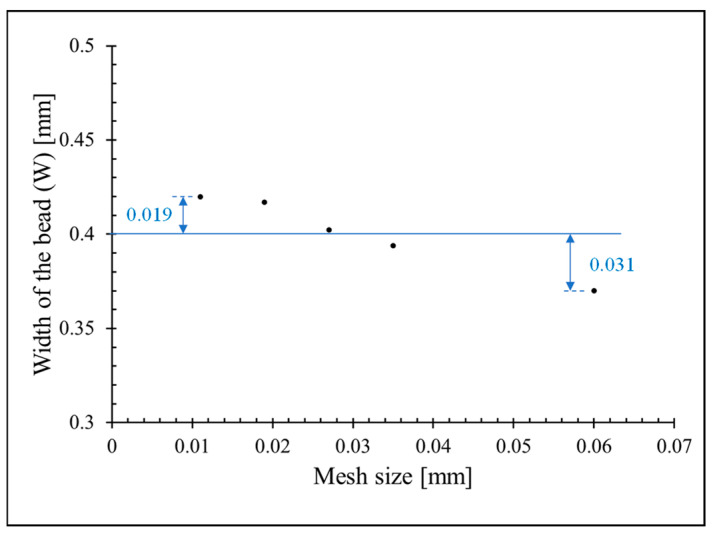
Influence of the mesh size on W.

Figure 5 represents the results of polymer deposition numerical simulation according to time for an example case (d_n_ = 0.4 mm, H_i_ = 0.4 mm, IV = 0.25 mm·s^−1^, and, TS = 20 mm·s^−1^). TPF numerical simulation is computationally demanding; consequently, the selection of the optimal mesh size is crucial for modeling. Fine mesh size highly increases the computation time while too coarse mesh size reduces the accuracy of the results. This is more notable in the case of TPF numerical simulation since the quality of the interface between air and polymer depends on the size of the mesh. Therefore, the size of the mesh must be optimized to get the fastest computation time while keeping the required accuracy of the obtained simulation. The mesh size selection for the 3D numerical simulation is more important compared with the 2D numerical simulation. In this study, tetrahedral mesh with a minimum 0.02 mm and maximum 0.06 mm mesh size is applied to the model. The mesh size is set finer near the nozzle and substrate compared with the air. The influence of the mesh size on W is represented in Figure 6. By decreasing the mesh size, W converges toward approximately 0.42 mm.

Two dimensionless numbers are defined to better study the influence of printing parameters on the deposited bead geometry. Equations (9) and (10) express the dimensionless number as the ratio between parameters related to the velocity (TS and IV) and geometry (H_i_ and d_n_).

(9) $$\pi_{1}=\frac{TS}{IV}$$

(10) $$\pi_{2}=\frac{Hi}{d_{n}}$$

### 2.3. Experimental Study

The experimental studies have been carried out using a PRUSA i3 MK3 RepRap (Feldkirchen, Germany) printer to validate the numerical simulation. In the RepRap printers, the IV of the filament in the nozzle is a function of extruder geometry (d_n_), TS, and filament characteristics, such as filament diameter (D). The polymer flow from the nozzle is controlled with the E-function in the generated G-code by slicing software, which corresponds to the length of the raw filament entering the extruder. Thus, it cannot be directly controlled by the users during the printing. Studying the influence of the polymer deposition velocity on the properties of the deposited bead is required to determine the IV of the polymer in the nozzle. Respectively, a series of experiments are conducted initially to spot the relation between the printing parameters, filament, and extruder geometry. The average IV of the filament in the nozzle is determined by using Equation (11) according to the printing parameters:

(11) $$\bar{IV}=\frac{E\times TS\times D^{2}}{L\times d_{n}^{2}}$$

where E (extruder increment) is the length of the filament entering the extruder for a given bead length. Note that the IV determined by Equation (11) is considered as mean or nominal velocity in the nozzle and L is the length of the deposited bead on the substrate.

For the experimental determination of the bead geometry, a single bead with a length (L) of 150 mm is deposited on a moving substrate through a nozzle diameter of the printer is 0.4 mm. For this study, the influence of H_i_, TS, and IV on the width of the bead is measured optically with a Leica (Wetzlar, Germany) DM2500M binocular with a 2× magnifier lens. The contact angle of the deposited bead with the substrate is measured experimentally using binoculars. The contact angle is independent of the printing parameters. The contact angle is considered as 30 degrees in this study.

## 3. Results and Discussion

This section focuses on analyzing the results of the numerical simulation and discussing the model validation. The contributions of this research to understanding the MEX process are summarized as follows: (1) determining the geometry of the deposited bead (extrudates) in the MEX process using LS approach numerical modeling; (2) determining the influence of the viscosity on the deposited bead using numerical modeling; and (3) comparing 2D and 3D numerical modeling. Therefore, a subsection is dedicated to each of these contributions.

Note that for the sake of consistency and conciseness, the machine parameter settings used in the case studies are reported in the form of a chain of the variable acronym (symbol) followed by the associated set values, without any space between parameters and values. For instance, d_n_0.4TS20IV25 indicates that d_n_ = 0.4 mm, TS = 20 mm·s^−1^, and IV = 25 mm·s^−1^.

### 3.1. Geometry of the Deposited Bead

The width of the deposited bead is a performance variable that is influenced directly by the printing parameter settings, machine accuracy, and other parameters defined in the slicing software. To determine the influence of printing parameters on the geometry of the deposited bead, the deposition of a single bead on a moving platform is modeled by numerical simulation and validated by experimental study. The influence of IV and TS on W is also determined by the experimental study for d_n_ = 0.4 mm and H_i_ = 0.3 mm. Table 3 summarizes the results of the bead width and compares the values from both simulation and experimental measurements. Table 3 also represents the deviation between the simulation and experimental measurements for each experiment. Note that, with the current machine configuration, the printing with TS/IV ratio above 1.25 is not feasible experimentally, hence these experiments are excluded from Table 3. Obtained results show an average error of 5.92% in predicting bead width.

**Figure 7:**
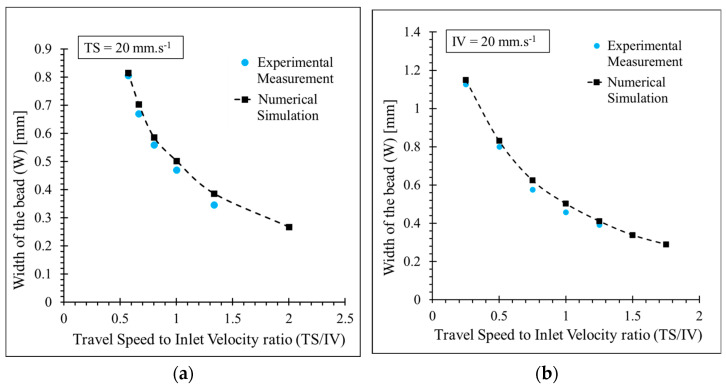
Comparison between the width of the bead (W) determined by numerical simulation and experimental study for d_n_ = 0.4 mm, H_i_ = 0.3 mm; (**a**) TS = 20 mm·s^−1^ and IV-variable, (**b**) TS-variable and IV = 20 mm·s^−1^.

The rest of the analysis is carried out in the following cases to evaluate the effect of IV and TS. For the first case, TS is considered constant (fixed at 20 mm·s^−1^) and IV is variable. For the second case, IV is fixed at 20 mm·s^−1^ and TS is variable. The ratio of TS to IV (π_1_) enables comparing these two cases. Figure 7 compares the numerical simulation and experimental study results for these two cases. For both cases, increasing the ratio of TS to IV (π_1_) leads to an increase in W. The obtained values for W found by numerical simulation have particularly good agreement with experimental studies, with an average error of 5.9% for the first series of experiments (first case) and an average error of 5.8% for the second series of experiments (second case). The small deviation between the expected and real value is mainly due to the uncertainty of the printed bead with a RepRap printer and systematic errors such as image measurement. Experimentally, it is not possible to determine W for TS to IV ratio (π_1_) above 1.25 due to poor contact of the deposited bead and platform and detachment of the bead during deposition. The same behavior is observed in the second case experiments. Poor contact between the deposited bead and the platform is also observed in the numerical simulation. The results of the numerical simulation and experimental study suggest that improving the adherence between the deposited bead and deposition platform requires decreasing TS and H_i_ and increasing IV. Superposing the two curves shown in Figure 7 reveals that the width of the bead is dependent on the π_1_ ratio and is independent of the value of TS or IV. This implies that TS and IV do not influence the bead’s geometry as long as the π_1_ is constant.

**Figure 8:**
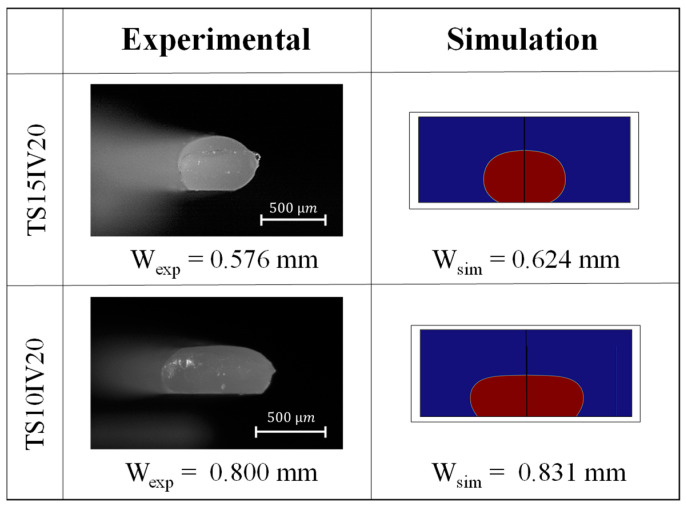
Comparison between the shape of the bead modeled by numerical simulation and experimental study.

Figure 8 represents the accordance of the results of numerical simulation and experimental study in predicting the shape of bead geometry for the two selected case studies.

**Figure 9:**
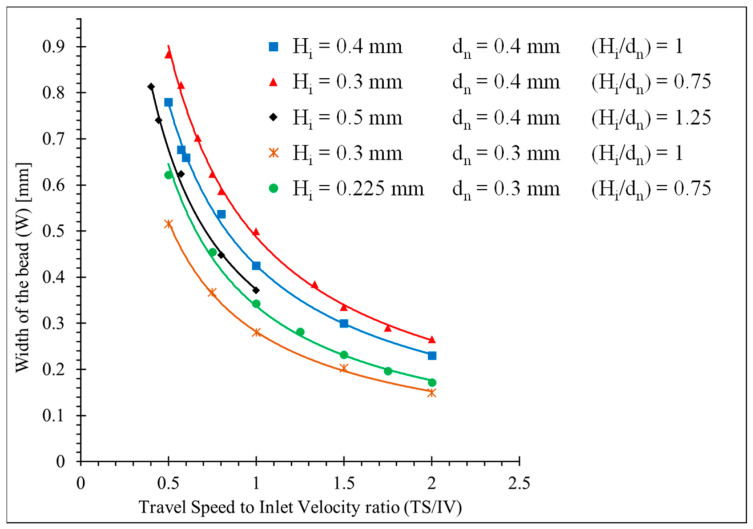
Determination of the width of the bead using numerical simulation.

Figure 9 illustrates the influence of the H_i_ and d_n_ on the width of the bead combined with TS and IV. Since d_n_ 0.3 and d_n_ 0.4 are commonly used in the MEX process, two levels were selected to determine the effect of d_n_ on W. The obtained results from the numerical simulation show that increasing d_n_ results in a wider bead while increasing H_i_ leads to a narrower bead. This is due to the insufficiency of the extruded material to fill the gap between the nozzle and the substrate. This results in the deterioration of the bead/substrate adherence and delamination of the layers. Therefore, a larger H_i_ and a smaller d_n_ are not recommended for the higher TS. As a rule of thumb, H_i_ should be equal to or smaller than d_n_, and the π_1_ ratio should be below one (1) when H_i_ is bigger than d_n_. Contrary to the π_1_ ratio, the same π_2_ ratio does not generate the same W. For instance, the red curve and green curve illustrated in Figure 9 have the same π_2_ value; however, they do not result in the same W.

### 3.2. Influence of Viscosity on the Geometry of the Deposited Bead

**Figure 10:**
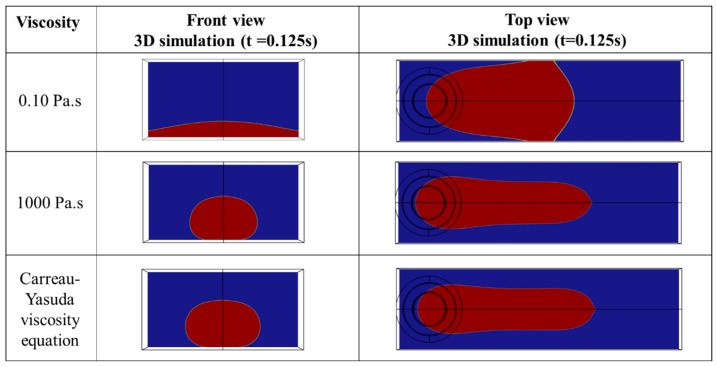
Influence of viscosity on the shape of the deposited bead (dn0.4IV0.25TS20).

This section investigates the influence of viscosity on the shape of the deposited bead by numerical simulation. Figure 10 shows the effect of different values of viscosity on the shape of the deposited bead. Three case studies, including the Carreau–Yasuda viscosity equation, constant viscosity below 0.1 Pa·s, and high constant viscosity of 1000 Pa·s, have been investigated. The molten polymer at low viscosity (e.g., 0.1 Pa·s) cannot keep its shape as a bead and eventually spreads on the substrate due to the gravity force. Increasing the viscosity above 10 Pa·s allows the polymer to keep its shape. It is observed that above 10 Pa·s shape of the deposited bead remains relatively constant, meaning that the shape of the deposited bead with 100 Pa·s and 1000 Pa·s is practically the same. This observation is in line with the numerical model developed by Comminal et al. [21]. The shape of the deposited bead with constant viscosity is compared with the model developed based on the Carreau–Yasuda viscosity represented in Equation (1). This comparison shows a minor difference between the shape of the deposited beads. According to the numerical simulation, the influence of viscosity on the geometry for the viscosity higher than 10 Pa·s is negligible.

The conclusion derived from Equations (2) and (3) indicates that the velocity field and shear rate in the nozzle are dependent on the IV, d_n,_ and n from the Carreau–Yasuda equation. Other parameters from the Carreau–Yasuda equation (η_inf_, η_0_, a, λ) do not influence the shear rate and velocity field in the nozzle. Furthermore, the influence of pseudoplasticity index (n) on the velocity field and shear rate is negligible compared with IV and d_n_. Consequently, the shape of the bead is independent of the amplitude of the viscosity during deposition. However, for the lower viscosities (less than 10 Pa·s), the applied gravity force on the deposited bead is greater than superficial forces and it causes the spread of polymer on the substrate. Furthermore, even in the case of considering the effect of pseudoplasticity index (n) by inserting viscosity as the Carreau–Yasuda equation, its influence is negligible on the velocity field and shear rate of the nozzle. When polymer melts exit from the nozzle, it is not in contact with the internal nozzle diameter, so immediately after exiting from the nozzle, the shear rate and velocity field are reduced toward zero (and viscosity is equivalent to the viscosity at the terminal regime). As a result, the shape of the bead is independent of the viscosity.

However, it is necessary to use the Carreau–Yasuda model to determine the shear rate in the nozzle and after deposition since the viscosity parameters (n and a) highly influence the shear rate. In addition to shear rate, viscosity influences the coalescence of the adjacent beads and layers [5]. Hence, it is essential to accurately determine the shear rate and, therefore, viscosity during the deposition.

### 3.3. Comparison between 2D and 3D Numerical Simulation

Numerical simulation of material deposition in 3D is computationally demanding and extremely sensitive to the accuracy of the boundary conditions definition and modeling. This motivates us to seek an alternative simpler modeling approach to measure the intended properties. This section aims to compare the modeling capability and accuracy of the 2D and 3D numerical simulations. The boundary conditions for 2D simulation are available in our previous publication [29]. Contrary to the 3D simulation, which allows the modeling of the height and width of the deposited bead in a single simulation, the modeling of height and bead in 2D requires two separate simulations.

**Figure 11:**
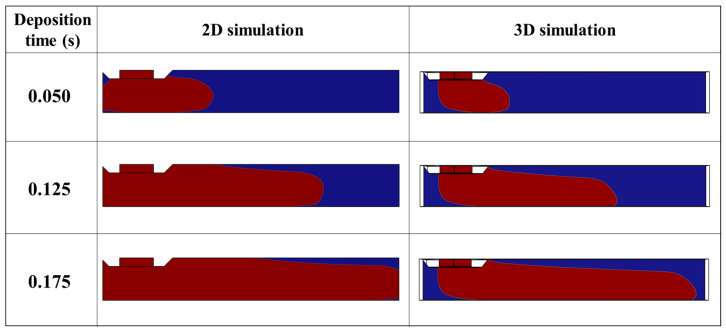
Comparison between 2D and 3D numerical simulation of material deposition at t = 0.125 s (dn0.4TS20IV25).

Due to this limitation of 2D simulation, the simulations are compared along the length of the bead by keeping the printing parameters constant. The results of the numerical simulations are visualized on a cross section in the XZ plane, which passes through the middle of the bead. Obviously, the 3D simulation is computationally more demanding than the 2D simulation, due to the increased number of mesh applied to the 3D model. The computational efficiency of 2D simulation allows us to define finer mesh sizes. The finer mesh size leads to a more accurate interface in 2D simulation compared with 3D simulation with a coarser mesh size. It is also possible to reduce the interfacial thickness parameter (ε_Is_) to make the interface even more accurate when the objective is to determine other properties such as heat transfer. In this study, a total of 14,284 triangle meshes with an average size of 0.0174 mm and a total number of 833,448 tetrahedra meshes with an average size of 0.0306 mm were used for 2D and 3D simulations, respectively. Despite the finer mesh size in 2D simulation, the 3D simulation took considerable time to be completed. It took approximately 12 h to complete 3D simulation and only around 1 h for 2D simulation using a desktop computer with the following specifications: Core i7-7700HQ CPU @ 2.80Hz and 32 GB RAM. The comparison between the shape of the bead modeled with 2D and 3D simulation reveals an enormous difference between the results, as shown in Figure 11. The simulation of the 2D model shows systematically more deposited material than the 3D model with the same parameter settings. This is mainly due to neglecting one of the dimensions in 2D simulation.

Even though 2D simulation is not suitable for predicting bead width, 2D simulations are still an effective approach in modeling parameters such as velocity field and shear rate.

Determination of properties such as transfer and kinetics of crystallization requires the simultaneous computation of several physics. Three-dimensional simulation is less effective for modeling these coupled properties since the model becomes too heavy to compute. In addition, lighter computational requirements for 2D simulations allow using finer mesh size in 2D simulation compared with 3D simulation. Using finer mesh size in 2D simulations leads to a more accurate interface near the interface of polymer and air. In the coupled simulations where several physics are involved, an inaccurate interface leads to deviation in the obtained results [29].

## 4. Conclusions

The presence of porosity in the printed parts is one of the main drawbacks of the MEX process. A high porosity ratio ultimately deteriorates the mechanical properties of the parts manufactured by the MEX process. In the MEX process, the porosity in the structure appears due to the shape of the adjacent beads during material deposition. Inconsistency in the bead size (width and height) also leads to an increase in porosity. Due to the deposition nature of the MEX process, the porosity cannot be entirely removed from the printed parts. However, understanding the influence of the printing parameters on the geometry of the deposited bead is essential to controlling the geometry of the deposited bead and adjusting it according to the desired geometry. Therefore, controlling the printing parameters is also essential in order to avoid excess material deposition to ensure uniformity of the width of the deposited bead. This is especially the case when new materials are developed and used in MEX processes. In the current work, the simulations are applied to PLA thermoplastic, which is widely used in 3D printing. However, the modeling approach can be applied to new materials developed in the field once the modeling approach is validated [33]. The contributions of this research to understanding the MEX process are as follows: (1) modeling the geometry of the deposited bead (extrudates) in the MEX process using LS approach numerical modeling; (2) investigation of the influence of the viscosity on the deposited bead using numerical modeling; and (3) comparison between 2D and 3D numerical modeling of the material deposition.

In this paper, two sets of experiments are carried out. In the first experimental step, the relation between the printing parameters and inlet velocity is identified. In the second set of experiments, the geometry of the bead is studied according to different printing parameters. The numerical simulations in this paper are used to investigate the influence of the printing parameters (layer height, travel speed, nozzle diameter, and polymer inlet velocity) and viscosity on the shape of the deposited bead. The width of the bead modeled with numerical simulation is compared and validated with the experimental results performed under the same conditions. Comparison between simulation results and experimental measurements indicates an average error of 5.92% in predicting the width of the deposited bead. Results obtained from numerical simulation reveal that while printing parameters highly influence the geometry, the shape of the deposited bead is independent of the viscosity. Experimental studies indicate the importance of the TS/IV ratio for the quality of the deposited bead. Deposition of the bead with a TS/IV ratio above 1.25 is experimentally impossible since it cannot properly adhere to the substrate (with the current machine configuration). However, numerical simulation could tackle this limitation. The results of 2D and 3D deposition modeling are compared. The observations reveal that 2D simulation is not appropriate and accurate enough to determine the geometry-dependent properties, such as the height of the bead. However, other printing properties, such as the shear rate, could be determined by 2D modeling. Future research will consist of modeling a matrix of deposited beads with different trajectory strategies based on known bead geometry. Another important research direction for a future contribution is to expand the model to study the influence of printing parameters on the shear rate of the deposited beads in the material extrusion process.

## Figures and Tables

**Table 1:** Values of the Carreau–Yasuda parameters for T = 195 °C.

Parameter (Symbol)	Value
Viscosity at infinity shear rate ($\eta_{inf})$	1945 ± 16 Pa·s
Viscosity at terminal regime ($\eta_{0})$	0 Pa·s
Relaxation time index (λ)	0.08 ± 0.02 s
Dimensionless viscosity transition index (a)	1.931 ± 0.5
Pseudoplasticity index (n)	0.693 ± 0.2

**Table 2:** Basic properties of the PLA used in this research.

Properties (Symbol)	Value
Filament diameter (D)	1.75 mm
Density (ρ)	1250 kg·m^−3^
Surface tension at melting state	0.028 N·m^−1^

**Table 3:** Comparison of bead width measurements between simulation and experiments.

No.	TS [mm·s^−1^]	IV [mm·s^−1^]	TS/IV [-]	W_sim_ [mm]	W_exp_ [mm]	Error [%]
1	5	20	0.25	1.150	1.127	2.08%
2	10	20	0.50	0.831	0.800	3.87%
3	15	20	0.75	0.624	0.576	8.40%
4	20	20	1.00	0.503	0.458	9.83%
5	25	20	1.25	0.412	0.392	5.05%
6	20	15	1.33	0.387	0.346	11.88%
7	20	20	1.00	0.503	0.470	7.02%
8	20	25	0.80	0.587	0.560	4.82%
9	20	30	0.67	0.704	0.670	5.13%
10	20	35	0.57	0.816	0.807	1.09%

## Data Availability

Data will be made available upon request.

## Nomenclature

MEX	Material extrusion process
PLA	Polylactic acid
ABS	Acrylonitrile butadiene styrene
PAEK	Polyaryl ether ketone
TFP	Two-phase flow
LS	Level-set
VOF	Volume-of-fluid
PF	Phase-field
CFD	Computational fluid dynamics
FFF	Fused filament fabrication
2D	Two dimension Two-dimensional al
3D	Three-dimensional
H_i_	Height of the layer [mm]
TS	Travel speed [mm·s^−1^]
IV	Inlet velocity [mm·s^−1^]
d_n_	Nozzle diameter [mm]
W	Width of the bead [mm]
L_i_	Initial length of the bead
D	Filament diameter [mm]
E	Extruder increment [mm]
a	Transition parameter in Carreau–Yasuda equation [1]
$\dot{\gamma }$	Shear rate [s^−1^]
$\eta_{0}$	Viscosity at terminal regime [Pa·s]
$\eta_{\inf}$	Viscosity at infinity shear rate [Pa·s]
ɛ_Is_	Interfacial thickness parameter [mm]
F	Other external forces [N]
F_st_	Surface tension force [kg⋅(m⋅s^−2^)]
g	Gravity acceleration [m·s^−2^]
n	Pseudoplasticity index [1]
T	Temperature [°C]
$\vec{u}$	Velocity field [mm·s^−1^]
P	Pressure [MPa]
γ	Re-initialization parameter [m·s^−1^]
ε	Coefficient of surface emissivity [1]
η	Viscosity [Pa·s]
η*	Complex viscosity [Pa·s]
μ	Surface tension [N·m^−1^]
ρ	Density [kg·m^−3^]
λ	Relaxation time index [s]
φ	Volume fraction [1]
T_s_	Temperature of substrate [°C]
T_c_	Temperature of printing chamber [°C]
T_p_	Printing temperature [°C]
T_b_	Temperature of the deposited bead [°C]
h	Coefficient of convection [W·(m·K)^−1^]
W_sim_	Width of a deposited bead from simulation [mm]
W_exp_	Sidth of a deposited bead from experiments [mm]
k	Coefficient of conduction [W·(m·K)^−1^]
$\pi_{1}$	Dimensionless number related to velocities [1]
$\pi_{2}$	Dimensionless number related to the dimensions [1]
